# Oleate promotes differentiation of chicken primary preadipocytes *in vitro*

**DOI:** 10.1042/BSR20130120

**Published:** 2014-02-07

**Authors:** Zhouchun Shang, Lin Guo, Ning Wang, Hui Shi, Yuxiang Wang, Hui Li

**Affiliations:** *Key Laboratory of Chicken Genetics and Breeding, Ministry of Agriculture, Harbin 150030, People's Republic of China; †Key Laboratory of Animal Genetics, Breeding and Reproduction, Education Department of Heilongjiang Province, Harbin 150030, People's Republic of China; ‡College of Animal Science and Technology, Northeast Agricultural University, Harbin 150030, People's Republic of China

**Keywords:** adipogenesis, chicken, differentiation, fatty acid, inducer, preadipocyte, AFABP, adipocyte fatty acid-binding protein, DMEM/F12, Dulbecco’s modified Eagle’s medium/Ham’s nutrient mixture F-12, GAPDH, glyceraldehyde-3-phosphate dehydrogenase, GATA2, GATA-binding protein 2, IBMX, isobutylmethylxanthine, IDX, insulin, dexamethasone and isobutylmethylxanthine, PPARγ, peroxisome proliferator-activated receptor-γ

## Abstract

In addition to providing energy and constituting cell membrane, fatty acids also play an important role in adipocyte differentiation and lipid metabolism. As an important member of monounsaturated fatty acids, oleate, together with other components, is widely used to induce chicken preadipocyte differentiation. However, it is not clear whether oleate alone can induce chicken preadipocyte differentiation. In the present study, four different treatments were designed to test this question: basal medium, IDX [insulin, dexamethasone and IBMX (isobutylmethylxanthine)], oleate and IDX plus oleate. Cytoplasmic lipid droplet accumulation and mRNA expression for adipogenesis-related genes were monitored. After treatment of oleate on chicken preadipocytes, apparent lipid droplet formation and lipid accumulation were observed, accompanied by increasing expression of PPARγ (peroxisome proliferator-activated receptor-γ) and AFABP (adipocyte fatty acid-binding protein), but decreasing level of GATA2 (GATA-binding protein 2). In contrast, for cells cultured in the basal medium with or without IDX supplementation, lipid droplet barely occurred. These results suggest that exogenous oleate alone can act as an inducer of preadipocyte differentiation into adipocytes.

## Abstract 2

Our results suggest that oleate alone can act as a direct inducer of chicken preadipocyte differentiation by elevating expression of key positive regulators and suppressing expression of negative regulator of adipogenesis.

## INTRODUCTION

Adipocyte differentiation has been extensively studied in mammalian preadipocyte cell lines, such as 3T3-L1 and 3T3-F442A cells [1,2]. These studies have revealed a complicated regulatory network of transcription factors underlies adipocyte differentiation. Apart from the adipogenesis-promoting factor PPARγ (peroxisome proliferator-activated receptor-γ), many anti-adipogenic transcription factors also play crucial roles in adipogenesis, including KLF7 (Kruppel-like factor 7) [3,4], KLF2 (Kruppel-like factor 2) [5] and GATA2 (GATA-binding protein 2) [6]. As for the molecular mechanism that controls adipogenesis, previous studies indicated that species-specific differences exist between birds and mammals [7]. A better understanding of the physiological characteristics and molecular events for chicken adipocyte differentiation will not only broaden our knowledge of adipocyte development across species, but also provide clues for reducing excessive fat deposition in chickens, which is one of the major complications in the modern broiler industry [8,9].

A widely employed adipogenesis-inducing agent in mammals is a mixture of hormones that consists of IDX [insulin, dexamethasone and IBMX (isobutylmethylxanthine)], which can induce preadipocyte differentiation by enhancing master regulators of adipocyte differentiation, and subsequent cytoplasmic lipid accumulation [6]. Since no chicken preadipocyte cell lines are available at present, most of the investigations of chicken preadipocyte differentiation are performed on primary stromal-vascular fraction cells derived from adipose tissues [4,10,11]. However, IDX mixture is insufficient in promoting chicken preadipocyte differentiation [12]. Several attempts have been made to optimize culture conditions for chicken adipocyte differentiation, such as the supplement of chicken serum, fatty acids, and hormone treatment as well [12,13]. Currently, fatty acids are thought to be an effective inducer of chicken preadipocyte differentiation [14].

Fatty acids are ubiquitous biological molecules. In addition to providing energy and constituting cell membrane to regulate physiological functions of cells and tissues, they can function as mediators of signal transduction and transcription for many genes [15,16]. Fatty acid mixtures (which contain a variety of long-chain fatty acids), but not dexamethasone, were found to be essential inducer of chicken adipocyte differentiation, by elevating PPARγ expression [14]. As an important member of fatty acids, oleate also plays a key role in adipocyte differentiation. Combined with hormones, oleate could definitely induce chicken primary preadipocytes into mature adipocytes [12]. Moreover, some researches showed that oleate possesses the ability to induce non-adipogenic cells to trans-differentiate into adipocytes solely. OA (oleic acid) exposure alone induced the intracellular lipid droplet accumulation and promoted the trans-differentiation to adipocytes of myoblast.[17]. Our previous study showed that OA can induce trans-differentiation of chicken fibroblasts into adipocyte-like cells [18]. To date, it is not clear whether oleate alone can induce chicken preadipocyte differentiation. To test this question, we compared the effects of IDX, OA and a combination of OA and IDX on chicken preadipocyte differentiation, and found that oleate could solely promote the differentiation of chicken primary preadipocyte.

## MATERIALS AND METHODS

### Primary culture of chicken preadipocytes

Chicken preadipocytes were cultured according to the methods as described in Ramsay [13], with some modifications. Abdominal adipose tissue was collected from 10-day-old broilers by sterile dissection following rapid decapitation. Adipose tissue was washed by pre-warmed PBS supplemented with penicillin (100 units/ml) and streptomycin (100 μg/ml), cut with surgical scissors, and digested in 2 mg/ml collagenase type I (Invitrogen) with shaking for 65 min at 37°C. After digestion, the cell suspension was filtrated through a 20-μm mesh and centrifuged at 300 ***g*** for 10 min at room temperature (22°C), to separate the stromal-vascular fractions from undigested tissue debris and mature adipocytes. Stromal-vascular cells (including preadipocytes) were seeded at a density of 5×10^4^ cells/ml in a basal medium [DMEM/F12 (Dulbecco's modified Eagle's medium/Ham's nutrient mixture F-12), 10% (v/v) FBS, 100 units/ml penicillin and 100 μg/ml streptomycin], and maintained in a humidified atmosphere with 5% (v/v) CO_2_ at 37°C until reaching confluence.

### Induction of chicken preadipocyte differentiation

**Figure 1:**
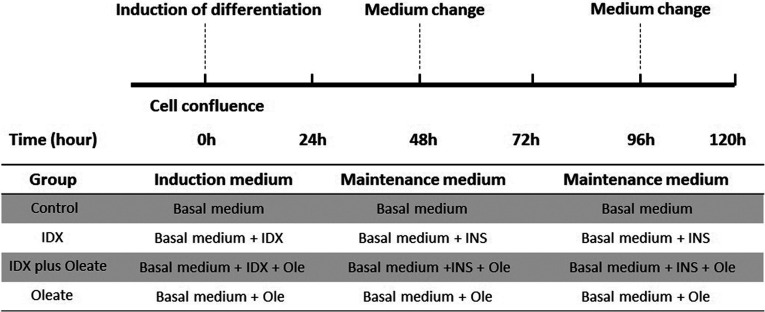
Overview of the experimental design in the study The media for the different treatments were changed at the indicated time points. The basal medium consisted of DMEM/F12 and 10% FBS. IDX consisted of insulin, dexamethasone and IBMX. The final concentration of oleate (Sigma) in the oleate group and the IDX plus oleate group was 160 μM. Further details were described as in the Materials and Methods section.

Following cell confluence, the medium for inducing differentiation was used, and changed every 2 days until day 5 of differentiation. Detailed procedures for the different treatments were described as in Figure 1. The basal medium was prepared using DMEM/F12 and 10% FBS. IDX was composed of 0.25 μM dexamethasone (Sigma), 10 μg/ml insulin (Sigma) and 0.5 mM IBMX (Sigma). Cells in the control group were cultured in the basal medium from 0 to 120 h. Cells in the oleate (Sigma) group were treated in the basal medium supplemented with oleate from 0 to 120 h. Cells in the IDX group were cultured in the basal medium supplemented with insulin, dexamethasone and IBMX at 0 h, and switched to insulin alone after 48 h, which was similar to mouse 3T3-L1 cells [19]. Cells in the IDX plus oleate group were cultured in the basal medium supplemented with insulin, dexamethasone, IBMX and oleate at 0 h, and switched to insulin and oleate after 48 h. The final concentration of oleate in the oleate group and the IDX plus oleate group was 160 μM.

### Lipid staining

Lipid droplets were stained by oil red O (Sigma) according to Yagi et al. [2], with some modifications. Cells were washed with PBS, and fixed with 10% (v/v) formalin in PBS at room temperature for 30 min. Then washed again with PBS, and stained with 0.5% oil red O-isopropyl alcohol solution for 8 min. After another wash with PBS, the cell nuclei were counterstained with Hoechst 33342 (Sigma). All experiments were repeated three times, and samples were treated in triplicate. Morphological changes were observed and photographed under an inverted fluorescent microscope (Leica).

### Measurement of lipid droplet accumulation

Lipid droplet accumulation was measured by oil red O extraction assay [20]. Cells in different groups were washed in PBS three times and fixed for 30 min with 10% formalin at room temperature. Then rinsed again with PBS, and stained with 1% oil red O staining solution [oil red O dye in 60% (v/v) isopropanol] for 40 min at room temperature. After removing the staining solution, oil red O was extracted by adding 1 ml of 100% (v/v) isopropyl alcohol, and measured at 500 nm using an ultraviolet spectrophotometer (Pharmacia). Adjacent plate wells with identical treatment were trypsinized, diluted and counted with a hemocytometer to normalize the extraction results.

### Quantification of gene expression using real-time PCR

Cells were harvested at 0, 12, 24, 48, 72, 96 and 120 h by removing the medium, and Trizol reagent (Invitrogen) was added directly into the culture dishes to isolate RNA. Total RNA was isolated and quantified using an ultraviolet spectrophotometer (Pharmacia) following the manufacturer's instructions. First-strand cDNA synthesis was performed with 1 μg total RNA (Promega A3500). To detect the expression of chicken adipogenesis-related genes, quantitative real-time PCR was performed using SYBR Premix Ex Taq (TaKaRa). Each RT-reaction was done in a 20-μl PCR reaction and the samples were incubated in the ABI 7500 real-time PCR system (Applied Biosystems) for an initial denaturation at 94°C for 15 s, followed by 40 cycles. Each cycle consisted of 94°C for 5 s and 60°C for 34 s. To confirm the amplification of the specific transcripts, melting curve profiles (cooling the sample to 65°C for 15 s and heating slowly to 94°C with continuous collection of fluorescence signal) were produced at the end of each PCR. The mRNA expression of PPARγ, AFABP (adipocyte fatty acid-binding protein) and GATA2 genes was calculated using the comparative 2^−Δ*C*^*^T^* method [21] with the GAPDH (glyceraldehyde-3-phosphate dehydrogenase) as an internal reference [22]. The sequences of the primers used in the experiment were shown as in Table 1.

**Table 1:** Primer sequences for real-time PCR

Gene name	Accession number	Primer sequence:5′–3′
PPAR-γ	AF470456	Sense GTGCAATCAAAATGGAGCC
		Antisense CTTACAACCTTCACATGCAT
AFABP	NM_204290	Sense ATGTGCGACCAGTTTGT
		Antisense TCACCATTGATGCTGATAG
GATA2	NM_001003797	Sense AACTGTGGAGCAACCGCTAC
		Antisense AGTCCGCAGGCATTACAAAC
GAPDH	K01458	Sense AGAACATCATCCCAGCGT
		Antisense AGCCTTCACTACCCTCTTG

### Statistical analysis

Results were given as mean±S.D. All data were subjected to one-way ANOVA analysis using SAS 9.13 software (SAS Institute), and differences at *P*<0.05 were considered significant.

## RESULTS

### Morphological changes in chicken preadipocytes

**Figure 2:**
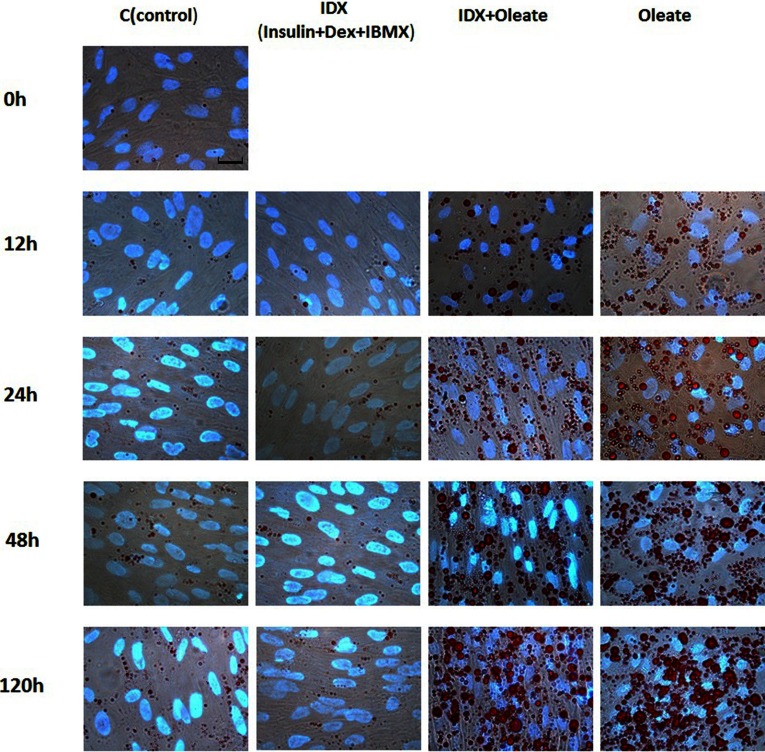
Morphological changes of chicken preadipocytes under different treatments Bar, 20 μm.

Cells were allowed to grow to confluence before administration of the treatment as described in Figure 1. Morphological changes during preadipocyte differentiation were monitored by oil red O staining at 0, 12, 24, 48 and 120 h for different treatments. Lipid droplets formed as early as 12 h, and lipid droplet accumulation were observed to be increasing during the subsequent culture period. Cells cultured with the oleate supplement showed a remarkable increase in the accumulation of lipid droplet, compared with those cultured in the basal medium with or without IDX at 48 and 120 h (Figure 2). Cells cultured in the basal medium or medium supplemented with IDX showed little lipid-droplet staining after reaching confluence (Figure 2).

### Lipid droplet formation and accumulation

**Figure 3:**
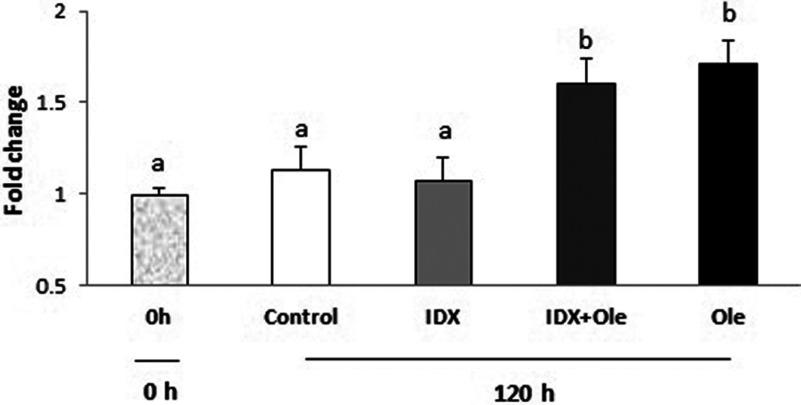
Intracellular lipid droplet accumulation in chicken preadipocytes under different treatments at 0 and 120 h The values for three replicates were given as the mean±S.D. Values with different alphabets (a, b) means significant differences between the time points (*P*<0.05). ole, oleate.

The formation and accumulation of lipid droplet were assessed by Oil Red O extraction assay at 0 and 120 h, after treatments with different inducing agents. At 120 h, chicken preadipocytes cultured in the medium supplemented with oleate alone showed significantly higher accumulation of lipid droplet compared with those in the basal medium with or without IDX (*P*<0.05) (Figure 3). These results suggest that oleate is potent in inducing the differentiation of chicken preadipocytes.

### Gene expression of adipogenesis-related genes

**Figure 4:**
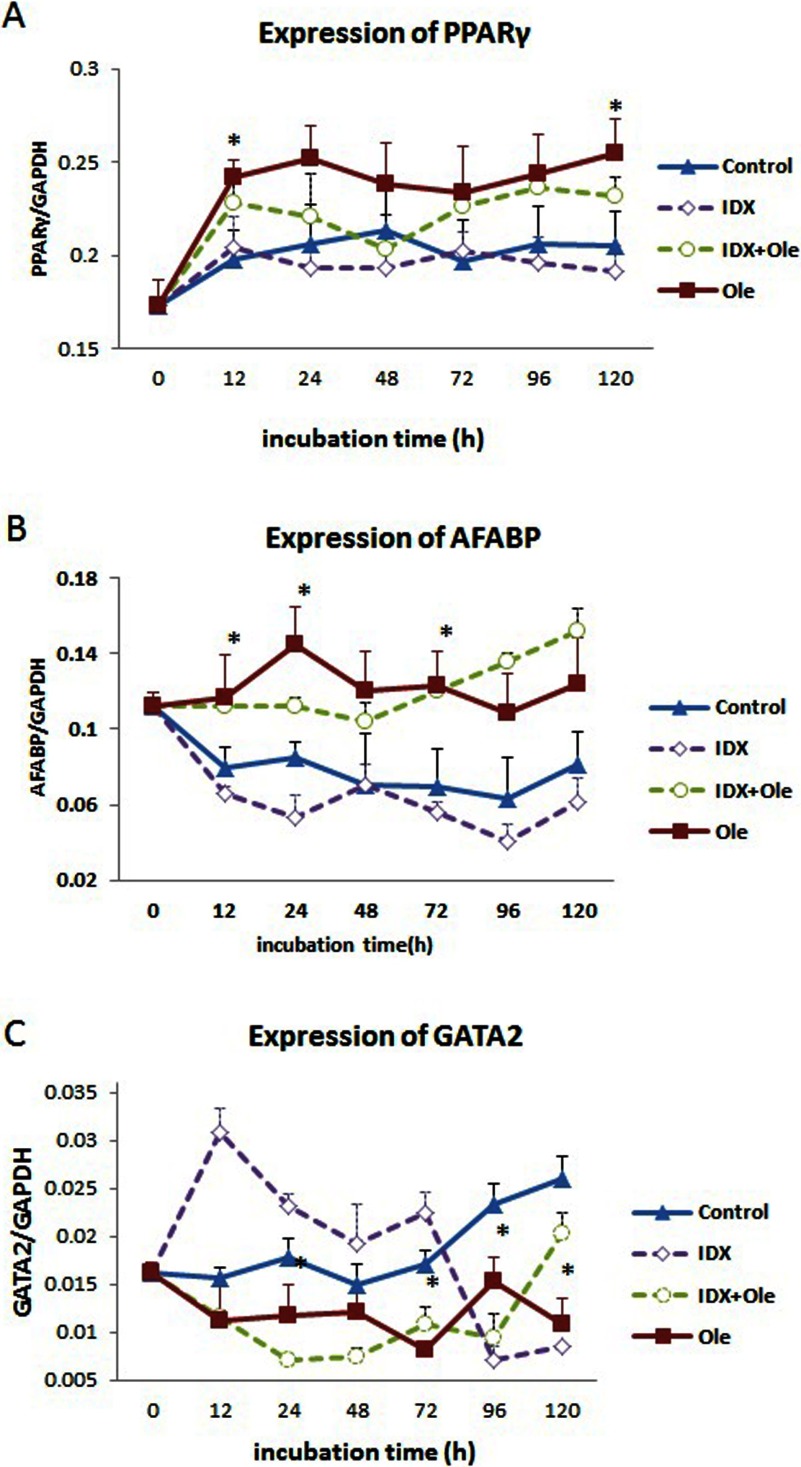
Expression of adipogenesis-related genes in chicken preadipocytes under different treatments The expression levels of mRNA were normalized to GAPDH, and expressed as a ratio of mRNA levels of genes of interest to that of GAPDH. The values of the three replicates are given as the mean±S.D. (A) PPARγ, (B) AFABP, (C) GATA2. *Significant differences between the oleate and control groups at the indicated time points (*P*<0.05).

Expression levels of PPARγ, AFABP and GATA2 genes were checked to examine the effects of the four different treatments on chicken preadipocyte differentiation. PPARγ mRNA expression in the oleate group gradually increased up to 24 h and maintained at a high level between 24 and 120 h. Significantly higher expression levels were observed at two time points, 12 and 120 h, compared with those in the control group, respectively. A similar expression pattern of PPARγ was also observed in the IDX plus oleate group. In comparison, the mRNA expression levels of PPARγ in the control and IDX groups remained at a low and unchanged level at all the six time points, from 12 until 120 h (Figure 4A).

As shown in Figure 4(B), AFABP mRNA expression in the oleate group clearly increased up to 24 h, followed by a relatively constant expression level between 24 and 120 h, and significantly higher expression levels were observed at 12, 24 and 72 h compared with those in the control group. Cells in the IDX plus oleate group also showed higher expression levels of AFABP between 12 and 120 h compared with those in the control and IDX groups.

In contrast, lower expression levels of GATA2 in the oleate and IDX plus oleate groups were observed at 12 and 120 h of culture, and significant differences between the oleate and control groups were observed at 24, 72, 96 and 120 h. Interestingly, expression of GATA2 in the IDX group showed relative higher levels at 12 and 24 h compared with the control group, and decreased sharply to lower levels at 96 and 120 h, respectively (Figure 4C).

## DISCUSSION

Previous studies have indicated that fatty acids, such as arachidonic acid [23], docosahexenoic acid [24] and eicosapentaenoic acid [25,26], can act as either promoters or inhibitors of mammalian adipocyte differentiation of preadipocyte cell lines. However, very few studies have addressed the effects of fatty acids on chicken adipocyte differentiation. Although chicken preadipocytes can be induced to differentiate into adipocytes by adding hormones and oleate or fatty acid mixtures [12,14], it is unclear whether chicken preadipocyte differentiation is induced by the combined effects of hormones and oleate, or due to the collective effects of fatty acid mixtures. Our results indicated that cells treated with oleate, and IDX plus oleate showed a marked increase in cytoplasmic lipid droplets, compared with the cells treated with IDX only. However, there was no significant difference in lipid droplet accumulation between oleate alone and oleate plus IDX groups (Figure 3). These results indicate that oleate alone can induce chicken preadipocyte differentiation. It is worth noting that cells in the control group also showed some formation and accumulation of lipid droplet (Figure 2), which indicated that chicken preadipocytes had a very low degree of spontaneous differentiation. This phenomenon was also observed in primary preadipocytes of many other species, probably caused by the complicated components in the FBS [27].

To further testify the pro-adipogenic effect of oleate on chicken preadipocyte differentiation, mRNA expression of PPARγ, AFABP and GATA2 for different treatments was monitored. PPARγ is the well-known master regulator of adipocyte differentiation [10,12,28]. Our data showed a high expression level of PPARγ mRNA from 12 and 120 h after oleate treatment. Similar observations were also obtained in chicken preadipocytes treated with fatty acid mixtures, in which PPARγ expression gradually increased during days 0–8 after induction of differentiation [14]. Taken together, these data suggest that oleate may promote chicken preadipocyte differentiation at least in part by activating the molecular programme controlled by PPARγ.

AFABP serves as an intracellular binding partner for long-chain fatty acids [29], and it can also function on ligand-dependent transactivation of PPARs by trafficking long-chain fatty acids to the nucleus [30]. The expression pattern of AFABP in the present study was elevated in the early stage of differentiation. This observation is consistent with those obtained in chicken preadipocytes induced by a combination of oleate and hormones, in which AFABP expression increased up to 24 h after induction [12].

Many anti-adipogenic transcription factors also play critical roles in adipogenesis. Forced expression of GATA2 in chicken primary preadipocytes suppressed its differentiation, and trapped cells at the preadipocyte stage [31]. In the present study, mRNA expression of GATA2 in the oleate group showed a tendency to decrease during differentiation (Figure 4C), suggesting that GATA2 also plays a negative role in chicken preadipocyte differentiation. Taken together, oleate induces chicken adipocyte differentiation, at least in part, by suppressing expression of GATA2.

The effect of IDX on chicken preadipocyte differentiation *in vitro* has not been well defined. Dexamethasone and IBMX have been shown to be insufficient in promoting chicken preadipocyte differentiation [13,14]. However, we could not exclude the effect of insulin on chicken preadipocyte differentiation, for previous study has indicated that the adipogenic effect of insulin on chicken preadipocytes might be compromised by high concentration of chicken serum [13]. In the present study, the basal medium contained 10% FBS, which might, to some extent, have also compromised the adipogenic effect of insulin on chicken preadipocyte differentiation.

In conclusion, oleate alone can act as a potent inducer of chicken preadipocyte differentiation. However, further investigation is still needed to clarify the molecular mechanisms underlying the pro-adipogenic effect of oleate on chicken preadipocytes.
