# Expression of Concern: Oleate promotes differentiation of chicken primary preadipocytes *in vitro*

**DOI:** 10.1042/BSR-20130120_EOC

**Published:** 2021-04-09

**Keywords:** adipogenesis, chicken, differentiation, fatty acid, inducer, preadipocyte

The Editorial Office has been made aware of potential issues surrounding the scientific validity of this paper, hence has issued an expression of concern to notify readers whilst the Editorial Office investigates.

